# GP IIb/IIIa-Mediated Platelet Activation and Its Modulation of the Immune Response of Monocytes Against *Candida albicans*

**DOI:** 10.3389/fcimb.2021.783085

**Published:** 2021-12-06

**Authors:** Lin Zheng, Zhimin Duan, Dingjie Tang, Yanzhi He, Xu Chen, Qing Chen, Min Li

**Affiliations:** ^1^ Institute of Dermatology, Jiangsu Key Laboratory of Molecular Biology for Skin Diseases and Sexually Transmitted Infections, Chinese Academy of Medical Sciences and Peking Union Medical College, Nanjing, China; ^2^ Jiangsu Province Blood Center, Nanjing, China; ^3^ School of Public Health, Nanjing Medical University, Nanjing, China; ^4^ Center for Global Health, School of Public Health, Nanjing Medical University, Nanjing, China

**Keywords:** *Candida albicans*, platelets, GP IIb/IIIa, PI3K/AKT, monocytes

## Abstract

*Candida albicans* is the most common fungal pathogen in humans, causing invasive disease and even potentially life-threatening systemic infections when tissue homeostasis is disrupted. Previous studies have identified an essential role of platelets in infection and immunity, especially when they are activated. However, it is still unclear whether platelets can be activated by *C. albicans*, and even less is known about the role of platelets in *C. albicans* infection. Herein, we showed that *C. albicans* induced platelet activation *in vitro*. *C. albicans* elevated the levels of AKT Ser473 phosphorylation, and inhibition of the PI3K-AKT signaling pathway reversed *C. albicans*-induced platelet activation. Surprisingly, *C. albicans*-induced platelet activation occurred in an integrin glycoprotein (GP) IIb/IIIa-dependent manner but was independent of the pattern recognition receptors toll-like receptor (TLR) 2 and TLR4. Interestingly, platelets enhanced the phagocytosis of human monocytes challenged with *C. albicans* and upregulated the expression of inflammatory cytokines, which were dependent on platelet activation mediated by GP IIb/IIIa. The present work provides new insights into the role of activated platelets in the defense against *C. albicans*, highlighting the importance of GP IIb/IIIa in the recognition of *C. albicans*.

## Introduction

*Candida* (*C.*) *albicans* is the most common fungal pathogen in humans, causing invasive disease and even potentially life-threatening systemic infections when tissue homeostasis is disrupted (Alby et al., 2009; Netea et al., 2015). *C. albicans* is also an emerging multidrug-resistant fungal pathogen and generates high healthcare costs worldwide (Dadar et al., 2018). Therefore, it is of great importance to understand how the body defends against *C. albicans* and find new therapeutic targets. Platelets are gradually recognized to be involved in infection, inflammation and immunity, in addition to their traditional role in mediating hemostasis and thrombosis (Semple et al., 2011; Yeaman, 2014). Several lines of evidence support a protective role for platelets in microbial infection: platelet microbicidal proteins (PMPs), kinocidins and defensins contained in platelet granules have direct antimicrobial effects (Tang et al., 2002; Yeaman, 2014); platelets and their products enhance the antimicrobial functions of immune cells by promoting phagocytosis, neutrophil extracellular trap (NET) formation and antigen presentation (Yeaman, 2014); and platelets phagocytize viruses or express the antiviral protein interferon-induced transmembrane protein 3 (IFITM3) to inhibit viral infection (Brass et al., 2009; Negrotto et al., 2015; Campbell et al., 2019).

Limited studies have explored whether platelets contribute to the host defense against *C. albicans*. Clinically, patients with thrombocytopenia had increased risks of candidiasis or candidemia (de Araujo Motta et al., 2016; Ekstrand et al., 2016), which suggested a potential role of platelets in the defense against *C. albicans*. Experimental evidence showed that activated platelets released PMPs such as CCL5 (also known as RANTES) and platelet factor 4 (PF4, also known as CXCL4), which exerted anti-*C. albicans* activity (Tang et al., 2002). In line with these findings, several reports have shown that activated platelets inhibit the growth of *C. albicans* (Willcox et al., 1998; Schultz et al., 2020). However, it is still unclear whether platelets can be activated by *C. albicans* and therefore promote its clearance by releasing anti-*C. albicans* products or other means. Previous reports have indicated that intracellular signaling pathways participate in a variety of platelet functions, including platelet activation, secretion and apoptosis (Chen et al., 2018; van der Meijden and Heemskerk, 2019). However, little is known about the role of signaling pathways in modulating the response of *C. albicans*-stimulated platelets.

Similar to innate immune cells, platelets express several classes of receptors that mediate the crosstalk between pathogens and platelets, including pattern recognition receptors, adhesion receptors and Fc receptors (Gautam et al., 2020). Glycoprotein (GP) IIb/IIIa, the most abundant integrin on platelet surfaces, plays a critical role in mediating platelet adhesion, activation, and aggregation (Gremmel et al., 2016; van der Meijden and Heemskerk, 2019). GP IIb/IIIa is also known to participate in pathogen recognition, such as that of specific viruses and bacteria, and to mediate platelet activation, virus internalization and thromboinflammation (Gautam et al., 2020). Whether GP IIb/IIIa is involved in mediating platelet recognition of *C. albicans* remains elusive.

Platelet interactions with monocytes-macrophages play a critical role in the response against bacterial infection by promoting macrophage polarization toward a proinflammatory phenotype (Carestia et al., 2019); enhancing capture, phagocytosis and intracellular killing of bacteria by macrophages (Wong et al., 2013; Ali et al., 2017; Carestia et al., 2019); and modulating inflammatory cytokine expression by monocytes and macrophages (Scull et al., 2010; Gudbrandsdottir et al., 2013; Xiang et al., 2013; Carestia et al., 2019). However, whether platelets interact with monocytes-macrophages in fungal infection has been poorly explored. In this regard, *in vitro* studies have shown the inhibition or induction of proinflammatory cytokines during coculture of platelets with monocytes-macrophages followed by stimulation with *Aspergillus* (*A.*) *fumigates* (Rodland et al., 2010; Czakai et al., 2017). Moreover, platelets have been found to enhance macrophage phagocytosis of *A. fumigates* (Czakai et al., 2017). Little is known about whether platelets interact with monocytes-macrophages during *C. albicans* infection. In the body, platelets most likely directly encounter *C. albicans* during candidemia and hence in the peripheral circulation. Therefore, we are more interested in whether platelets interact with monocytes in response to *C. albicans* rather than with macrophages.

In this study, we show that *C. albicans* induces platelet activation *in vitro*. We demonstrate that *C. albicans*-induced platelet activation occurs in a GP IIb/IIIa-dependent manner, independent of toll-like receptor (TLR) 2 and TLR4. The PI3K-AKT signaling pathway mediates *C. albicans*-induced platelet activation. Platelets enhance phagocytosis of human monocytes challenged with *C. albicans* and upregulate the expression of inflammatory cytokines, which are dependent on platelet activation mediated by GP IIb/IIIa. The present work provides new insights into the role of activated platelets in the defense against *C. albicans*, highlighting the importance of GP IIb/IIIa in the recognition of *C. albicans*.

## Materials and Methods

### *C. albicans* Cultivation and Staining

*C. albicans* strain SC5314 (from the China Medical Fungus Culture Collection Center) was grown for 12 h at 30°C in yeast extract-peptone-dextrose (YPD) broth. *C. albicans* was then collected by centrifugation at 2000×*g* for 5 min. Following centrifugation, *C. albicans* was resuspended in 30 mL of phosphate-buffered saline (PBS) and washed twice. Yeast cells were enumerated using a hemocytometer and then diluted with PBS to the desired working concentrations. Before *C. albicans* stimulated platelets, *C. albicans* was washed with PBS to remove their supernatant. In some experiments, after being washed and diluted with PBS, *C. albicans* was heat killed immediately in an 85°C water bath for 10 min. Except when otherwise specified, live microorganisms were used in the experiments. FUN-1 (Thermo Fisher, Waltham, MA, USA, F7030) staining of *C. albicans* was used for flow cytometry. The staining solution was added at a dilution of 1:1000 (v/v) to *C. albicans* and incubated for 30 min, followed by washing with PBS and then diluting to the desired working concentrations.

### Platelet Stimulation

Platelet-rich plasma (PRP) was prepared as concentrates by thrombocytapheresis with an Amicus cell separator (Baxter) by the Department of Blood Transfusion (Jiangsu Province Blood Center). Platelet counts were performed with a Sysmex poch-80i hematologic analyzer (Sysmex Corporation). For washed platelet suspensions, PRP was washed twice with CGS buffer (0.123 M NaCl, 0.033 M D-glucose, 0.013 M trisodium citrate, pH 6.5). Platelets were collected by centrifugation at 1100×*g* for 5 min. Then platelets were resuspended in Medium 199 (Gibco, NY, USA, 12340030) and kept at room temperature until the experiment was performed. Except when otherwise specified, PRP was used in the experiments. To obtain platelet-poor plasma (PPP), PRP was centrifuged at 1500×*g* for 5 min to remove platelets.

PRP was incubated with heat-killed (HK) *C. albicans* spores for 6 h (1 h, 3 h, or 6 h in the western blotting experiments) or live *C. albicans* spores for 2 h at a multiplicity of infection (MOI) of 0.01 at 37°C. PRP incubated with equal volume of PBS was used as control. For the signaling pathway inhibition experiments, platelets were pretreated with the PI3K inhibitor LY294002 (Selleckchem, Houston, TX, USA, S1105, 90 μM) and Akt inhibitor MK2206 (Selleckchem, S1078, 30 μM) or vehicle control at 37°C for 2 h and then were incubated with live *C. albicans* at 37°C for 2 h. For the receptor inhibition experiments, platelets were costimulated with *C. albicans* after pretreatment with the GP IIb/IIIa-specific antagonist tirofiban (Selleckchem, S8594, 100 or 200 μg/mL), anti-TLR2 neutralizing antibody (*In vivo*Gen, San Diego, CA, USA, maba2-htlr2, 1, 5, or 20 μg/mL), anti-TLR4 neutralizing antibody (*In vivo*Gen, mabg-htlr4, 1, 5, or 20 μg/mL) or vehicle control (*In vivo*Gen, maba2-ctrl, mabg1-ctrlm, 1, 5, or 20 μg/mL, respectively) at 37°C for 45 min. For the GP IIb/IIIa receptor blocking experiments, 2 × 10^7^ washed platelets were pretreated with the anti-GP IIb/IIIa antibody (BioLegend, San Diego, CA, USA, 359804, clone A2A9/6) at 12.5 μg/mL or vehicle control (BioLegend, 401507) at 37°C for 30 min and then incubated with live *C. albicans* at 37°C for 2 h. For the agonist experiments, platelets were stimulated with different concentrations of the TLR2 agonist Pam3CSK4 (*In vivo*Gen, tlrl-pms, 2, 10, or 25 μg/mL), FSL-1 (*In vivo*Gen, tlrl-fsl, 0.1, 0.5, or 1 μg/mL), and the TLR4 agonist LPS (Sigma-Aldrich, St. Louis, MO, USA, L2880, 1, 5, or 25 μg/mL) at 37°C for 6 h.

### Preparation and Stimulation of THP-1 Cells and Primary Human Monocytes

Human monocytic leukemia cell line, THP-1, was purchased from the American Type Culture Collection (ATCC, Manassas, VA, USA, TIB-202). Peripheral blood mononuclear cells (PBMCs) were isolated from 3 healthy volunteers (3 biological replicates) by Lymphoprep (STEMCELL Technologies, Vancouver, BC, Canada). To isolate primary human monocytes, magnetic activated cell sorting with paramagnetic CD14-beads (Miltenyi Biotec, Paris, France) was used to separate CD14^+^ monocytes according to the manufacturer’s instructions. THP-1 cells and primary human monocytes were cultured in complete medium [RPMI 1640 (Gibco) containing 10% fetal bovine serum (Gibco), 100 U/mL penicillin (Gibco), and 100 mg/mL streptomycin (Gibco)] in a humidified incubator with 5% CO_2_ at 37°C. For stimulation experiments, THP-1 cells and primary human monocytes were seeded in 6-well plates at a density of 1 × 10^6^ cells/well and then cocultured with *C. albicans* (3:1, fungi to monocytes) in the presence of PRP (50:1, platelets to monocytes) or an equal volume of PPP at 37°C for 2 h. For inhibitor experiments, PRP was pretreated with tirofiban (200 μg/mL) or the antiplatelet agent cilostazol (Selleckchem, S1294, 200 μM) for 45 min before costimulation with THP-1 cells and *C. albicans*.

### Flow Cytometry

To analyze platelet activation, cultured platelets were centrifuged at 1200×*g* for 5 min at 4°C and fixed with 1% paraformaldehyde on ice for 30 min. Then, platelets were washed with PBS once and diluted to 10^6^/100 μL. Platelets were stained with 1:200-diluted CD41a-phycoerythrin (PE)-Cy7 (Thermo Fisher, 25-0419-42, clone HIP8) and 1:150-diluted P-selectin-APC (Thermo Fisher, 17-0626-82, clone Psel.K02.3) or 1:150-diluted immunoglobulin isotype control of P-selectin-APC (Thermo Fisher, 17-4714-82, clone P3.6.2.8.1), followed by incubation for 30 min at room temperature. After washing, the samples were assessed immediately on a flow cytometer (BD FACSVers). The expression of GP IIb/IIIa (also known as CD41/CD61) on platelets was identified using CD41a-PE-Cy7. To assess the phagocytosis of *C. albicans* by THP-1 cells or primary human monocytes, cultured cells were collected into flow tubes and centrifuged at 300×*g* for 5 min, followed by washing with ice-cold PBS to clear nonphagocytosed *C. albicans* and platelets. Then, samples were assessed on a flow cytometer. Untreated THP-1 cells or primary human monocytes were used as a negative control, and pure FUN-1-*C. albicans* was used as a positive control, with gating according to forward and side scatter. Phagocytosis of *C. albicans*-FUN-1 by THP-1 cells or primary human monocytes was assessed based on the fluorescence of the FITC channel.

### Enzyme-Linked Immunosorbent Assay (ELISA)

PRP from a single donor was paired to be treated with either *C. albicans* or PBS. After costimulation of platelets and *C. albicans*, platelets were centrifuged at 1500×*g* for 5 min at 4°C. Cell supernatant was collected and stored immediately at -80°C until cytokines were measured. The levels of PF4 (RayBiotech, Norcross, GA, USA), CCL5 (R&D Systems, Minneapolis, MN, USA), β-defensin-2 (Arigo Biolaboratories, Hsinchu, China), tumor necrosis factor (TNF) -α (R&D Systems), interleukin (IL) -1β (R&D Systems), and CXCL8 (R&D Systems) were measured using commercial ELISA kits according to the manufacturer’s instructions.

### Western Blotting

Cultured platelets were washed twice with ice-cold PBS and then lysed with RIPA buffer (CWBIO, Beijing, China) containing protease and phosphatase inhibitors (Roche, Mannheim, Germany) on ice for 20 min. After centrifugation (12,000×*g*, 4°C for 5 min), the supernatant was collected. The protein concentration was evaluated using a Pierce BCA Protein Assay Kit (Thermo Fisher, 23227). Then, SDS (Generay Biotech, Shanghai, China) and RIPA buffer were added to the solution, followed by boiling for 5 min. Antibodies against p-Stat3 (1:1000, Cell Signaling Technology, Danvers, MA, USA, 9145), Stat3 (1:1000, Cell Signaling Technology, 4904), p-AKT (1:1000, Cell Signaling Technology, 4060), AKT (1:1000, Cell Signaling Technology, 4691), p-ERK1/2 (1:1000, Cell Signaling Technology, 4377), ERK1/2 (1:1000, Cell Signaling Technology, 4695), p-p38 (1:1000, Cell Signaling Technology, 4511), p38 (1:1000, Cell Signaling Technology, 9212), and GAPDH (1:1000, Cell Signaling Technology, 5174) were used for immunoblot analysis according to the manufacturers’ protocols. Proteins were separated by 10% SDS-polyacrylamide gels (Beyotime, Shanghai, China) and visualized by the ECL system. After detection of phosphorylated proteins, the membrane was stripped (Thermo Fisher, 46430) for 15 min on a rotator before blotting for total protein. Quantification was performed with ImageJ software.

### RNA Extraction and Quantitative Real-Time PCR

Total RNA was extracted from cultured cells using RNAiso Plus reagent (Takara, Tokyo, Japan). For each well, approximately 8 µg of RNA was yielded. Complementary DNA was synthesized using 500 ng with Prime Script RT Master Mix (Takara). Quantitative PCR (qPCR) was carried out with iTaq Universal SYBR Green Supermix (Bio-Rad, Hercules, CA, USA) and 7300 System SDS Software (version 1.4.0, Applied Biosystems). The relative expression of target genes was calculated through the 2-ΔΔCT method relative to the reference gene (GAPDH), and then, the fold change relative to the reference sample was calculated. The following primers were used:

GAPDH (human) forward: 5′-GTCTCCTCTGACTTCAACAGCG-3′, reverse: 5′-ACCACCCTGTTGCTGTAGCCAA-3′; IL-1β (human) forward: 5′-CCACAGACCTTCCAGGAGAATG-3′, reverse: 5′-GTGCAGTTCAGTGATCGTACAGG-3′; IL-6 (human) forward: 5′-TTCGGCAAATGTAGCATG-3′, reverse: 5′-AATAGTGTCCTAACGCTCATAC-3′; TNF-α (human) forward: 5′-CTCTTCTGCCTGCTGCACTTTG-3′, reverse: 5′-ATGGGCTACAGGCTTGTCACTC-3′; and CXCL8 (human) forward: 5′-TTTTGCCAAGGAGTGCTAAAG-3′, reverse: 5′-AACCCTCTGCACCCAGTTTTC-3′.

### Statistical Analysis

The statistical tests used are specified in the figure legends. Numeric data were analyzed using one-way analysis of variance (ANOVA). Two groups were compared by a two-tailed Student’s t test. The significance of the data was assessed using GraphPad Prism version 8.0 (GraphPad Software, San Diego, CA, USA). A *P*-value less than 0.05 was considered statistically significant.

## Results

### *C. albicans* Induces Platelet Activation

**Figure 1:**
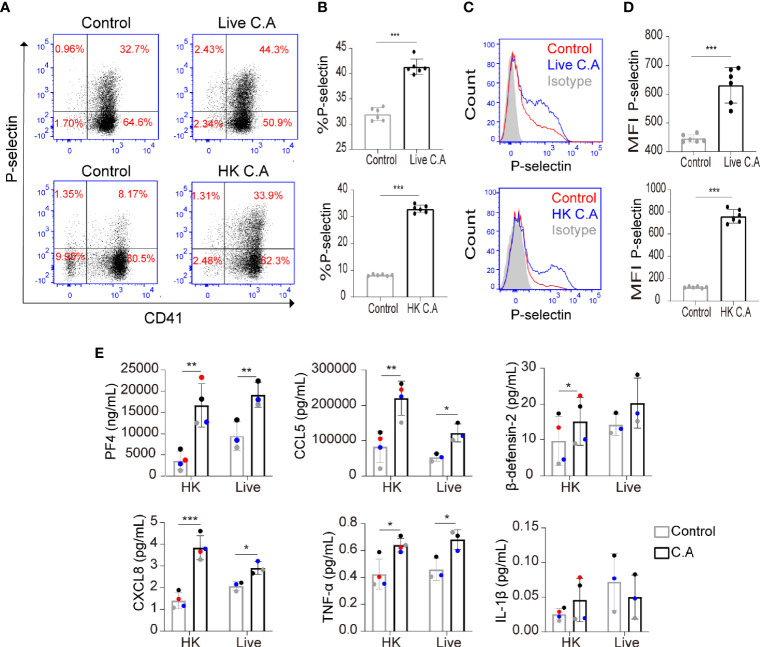
*C. albicans* induces platelet activation. **(A–E)** Platelet-rich plasma (PRP) was incubated with live *C. albicans* (**A–D**, top column) for 2 h or heat-killed (HK) *C. albicans* (**A–D**, bottom column) for 6 h at a multiplicity of infection (MOI) of 0.01. PRP incubated with equal volume of PBS was used as control. Cells were fixed, stained for the general platelet marker CD41 and the activation marker P-selectin and analyzed using flow cytometry. Representative dot plots **(A)** and flow cytometry histograms **(C)** are shown. The percentages **(B)** and mean fluorescence intensity (MFI) **(D)** of P-selectin-positive cells were calculated. **(E)** PRP from a single donor was paired to be treated with either *C. albicans* or PBS. Levels of PF4, CCL5, β-defensin-2, CXCL8, TNF-α and IL-1β in coculture suspension were determined by enzyme-linked immunosorbent assay (ELISA). The two data points from a single donor were presented with the same color. Data represent two or more independent experiments. Data are expressed as the mean ± SD determined by unpaired **(B, D)** or paired **(E)** Student’s t test; *P < 0.05, **P < 0.01, ***P < 0.001. C.A, *C. albicans*; HK, heat-killed.

To investigate whether *C. albicans* could induce platelet activation, we incubated live *C. albicans* and HK *C. albicans* with human platelets and evaluated the expression of P-selectin (also known as CD62P) on platelets, which translocates from α-granules to the platelet surface membrane following platelet activation (Blair and Flaumenhaft, 2009). We found that both live *C. albicans* and HK *C. albicans* significantly increased the expression of P-selectin on platelets (**Figures 1A–D**). To exclude the influence of experimental settings on platelet activation, we also incubated *Candida parapsilosis* (*C. parapsilosis*) and *Cutibacterium acnes* (*C. acnes*) with platelets. We found that *C. acnes* could not increased the expression of P-selectin on platelets. Only HK *C. parapsilosis* but live *C. parapsilosis* could increase the expression of P-selectin. The expression of P-selectin on HK *C. parapsilosis*-treated platelets was less than that on HK *C. albicans*-treated platelets (**Supplemental Figure S1**). Generally, activated platelets secrete a variety of chemokines, including PF4 and CCL5. PF4 is also regarded as a marker of platelet activation (Gremmel et al., 2016). We found that both live *C. albicans* and HK *C. albicans* significantly elevated the levels of PF4 and CCL5 in the coculture suspension (**Figure 1E**).

The inflammatory cytokines TNF-α, IL-1β, and CXCL8 and the antimicrobial peptide β-defensin-2 play an important role in resisting *C. albicans* and have been reported to be released by activated platelets (Lindemann et al., 2001; Aslam et al., 2006; Tohidnezhad et al., 2012; Yeaman, 2014; Netea et al., 2015; Jarva et al., 2018; Fusco et al., 2021). Therefore, we further determined whether *C. albicans* enhanced the secretion of these proteins by platelets. We found that both live *C. albicans* and HK *C. albicans* significantly elevated the secretion of low concentrations of CXCL8 and TNF-α from platelets (**Figure 1E**). HK *C. albicans* elevated the secretion of β-defensin-2, while live *C. albicans* did not (**Figure 1E**). The level of IL-1β, which was secreted at a relatively low concentration, did not vary in HK *C. albicans*-treated or live *C. albicans*-treated platelets (**Figure 1E**).

### *C. albicans* Induces Platelet Activation Through the PI3K-AKT Signaling Pathway

**Figure 2:**
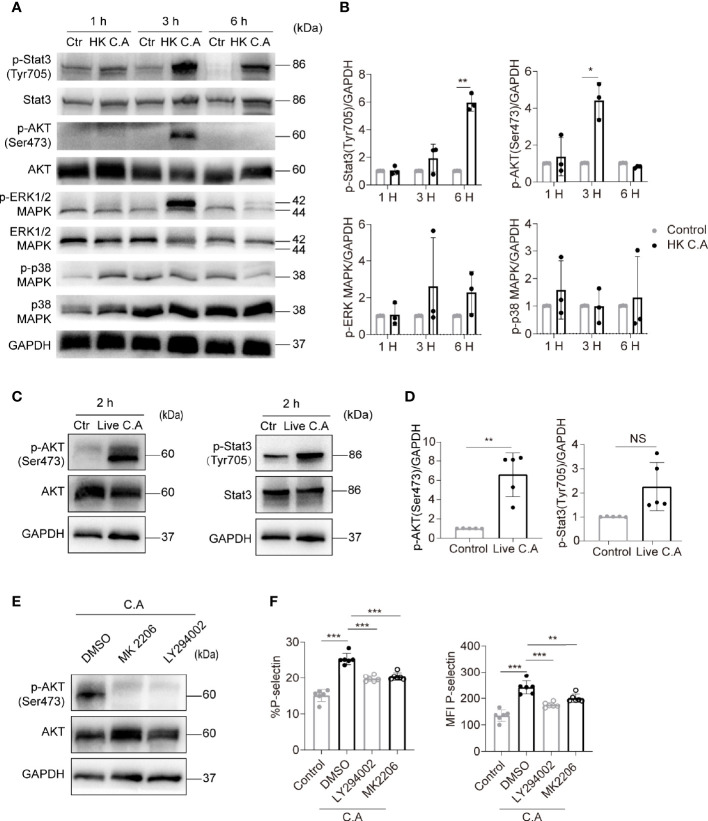
*C. albicans* induces platelet activation through the PI3K-AKT signaling pathway. **(A–D)** Western blot analysis of protein levels in human platelets treated with HK *C. albicans* **(A, B)** for 1 h, 3 h, or 6 h or live *C. albicans* **(C, D)** for 2 h at an MOI of 0.01. PRP incubated with equal volume of PBS was used as control. The data are representative of three or more independent experiments. **(E, F)** Human platelets were pretreated with the PI3K inhibitor LY294002 (90 μM), Akt inhibitor MK2206 (30 μM), or vehicle control at 37°C for 2 h and then incubated with live *C. albicans* at 37°C for 2h. Western blot analysis of Akt phosphorylation with anti-pAkt Ser-473 antibodies is shown **(E)**. The expression of P-selectin was detected by flow cytometry. The percentages (F left column) and MFI (**F** right column) of P-selectin-positive cells were calculated. Data are expressed as the mean ± SD; *P < 0.05, **P < 0.01, ***P < 0.001, compared with the vehicle control *via* Student’s *t* test. C.A, *C. albicans*; Ctr, control; HK, heat-killed; NS, not significant.

Platelet activation requires intracellular signal transduction initiated by pathogen- and damage-associated molecular pattern molecules, adhesion proteins and soluble agonists (Gremmel et al., 2016; Estevez and Du, 2017). Next, we investigated the mechanism of *C. albicans*-induced platelet activation. Stat3, AKT, ERK1/2 and p38 phosphorylation have been shown to be involved in platelet signal transduction when platelets are activated (Rex et al., 2009; Zhou et al., 2013; Guidetti et al., 2015). Therefore, we detected the phosphorylation of these proteins to further explore the signaling pathway mediating *C. albicans*-induced platelet activation. We found that AKT phosphorylation on Ser473 and Stat3 phosphorylation on Tyr705 were elevated in HK *C. albicans*-treated platelets (**Figures 2A, B**). However, only AKT Ser473 phosphorylation levels were significantly elevated in live *C. albicans*-treated platelets (**Figures 2C, D**).

The PI3K-Akt signaling pathway has been deeply investigated in platelet activation (Guidetti et al., 2015). Therefore, we hypothesized that the PI3K-Akt pathway mediated *C. albicans*-induced platelet activation. To test this hypothesis, platelets were treated with the PI3K inhibitor LY294002 and Akt inhibitor MK2206 before costimulation with *C. albicans*. LY294002 and MK2206 markedly reduced AKT Ser473 phosphorylation levels (**Figure 2E**) and P-selectin expression on platelets (**Figure 2F**). These data suggest that *C. albicans* induces platelet activation through a PI3K-AKT signaling pathway.

### *C. albicans* Induces Platelet Activation in a GP IIb/IIIa-Dependent Manner

As mentioned above, platelets express pattern recognition receptors, such as TLR2 and TLR4, and these receptors recognize a variety of microbes and hence facilitate platelet activation, platelet-neutrophil aggregation, and leukocyte phagocytosis (Schattner, 2019; Gautam et al., 2020). To explore whether TLR2 and TLR4 mediated *C. albicans*-induced platelet activation, we treated platelets with anti-TLR2 or anti-TLR4 neutralizing antibodies before costimulation with *C. albicans*. We found that blocking TLR2 or TLR4 did not suppress the expression of P-selectin (**Supplemental Figure S2A**). In addition, platelet stimulation with the TLR2 agonists Pam3CSK4 and FSL-1 and the TLR4 agonist LPS did not elevate the expression of P-selectin (**Supplemental Figure S2B**) or secretion of PF4 (**Supplemental Figure S2C**) as *C. albicans* did. These data suggest that *C. albicans*-mediated platelet activation is independent of the TLR2 and TLR4 receptors.

**Figure 3:**
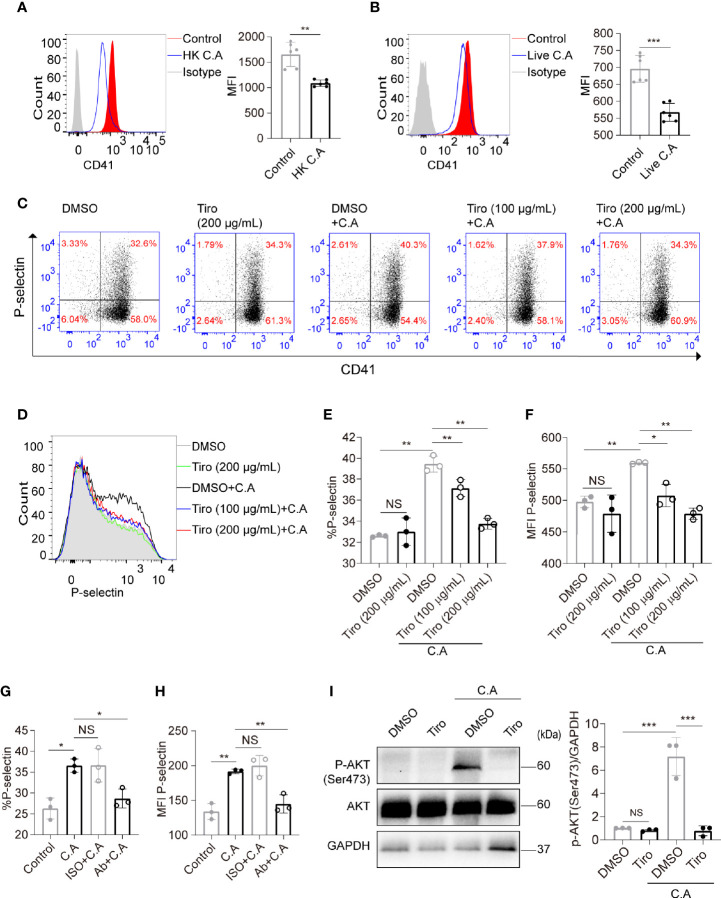
*C. albicans* induces platelet activation in a GP IIb/IIIa-dependent manner. **(A, B)** Platelets were incubated with HK *C. albicans* **(A)** for 6 h or live *C. albicans* **(B)** for 2 h at an MOI of 0.01. The expression of GP IIb/IIIa (CD41/CD61) was detected by flow cytometry using a PE-Cy7-conjugated anti-CD41a antibody. **(C–F)** Platelets were pretreated with the GP IIb/IIIa-specific antagonist tirofiban at 100 μg/mL, tirofiban at 200 μg/mL or vehicle control at 37°C for 45 min and then incubated with live *C. albicans* at 37°C for 2h. Representative dot plots **(C)** and flow cytometry histograms **(D)** are shown. The percentages **(E)** and MFI **(F)** of P-selectin-positive cells were calculated. **(G, H)** Washed platelets were pretreated with the blocking antibody for GP IIb/IIIa (Ab) at 12.5 μg/mL or vehicle control (ISO, isotype) at 37°C for 30 min and then incubated with live *C. albicans* at 37°C for 2 h. The percentages **(G)** and MFI **(H)** of P-selectin-positive cells were calculated. **(I)** Platelets were pretreated with tirofiban (200 μg/mL) or vehicle control at 37°C for 45 min and then incubated with *C. albicans* at 37°C for 2h. Western blot analysis of Akt phosphorylation with anti-pAkt Ser-473 antibodies. Data are expressed as the mean ± SD; *P < 0.05, **P < 0.01, ***P < 0.001 compared with the control by Student’s t test **(A, B)** or one-way analysis of variance (ANOVA) followed by Bonferroni’s *post hoc* test **(E–I)**. Ab, anti-GP IIb/IIIa antibody; C.A, *C. albicans*; HK, heat-killed; ISO, isotype; NS, not significant; Tiro, tirofiban.

GP IIb/IIIa is capable of mediating platelet activation and PI3K-Akt pathway activation when recognizing specific pathogens or nonpathogen ligands (Guidetti et al., 2015; Gautam et al., 2020). Therefore, we further explored whether GP IIb/IIIa mediated *C. albicans*-induced platelet activation. Surprisingly, we found that the expression of GP IIb/IIIa determined by flow cytometry was reduced on *C. albicans*-treated platelets (**Figures 3A, B**). We speculated that *C. albicans* bound to the GP IIb/IIIa receptor and thus reduced its detectable amounts. We also found that the binding of FITC-coupled antibody PAC-1, which bound only to the activated form of GP IIb/IIIa, did not vary on *C. albicans*-treated and control platelets (**Supplemental Figure S3**).

To further clarify the role of GP IIb/IIIa in *C. albicans-*induced platelet activation, we pretreated platelets with the GP IIb/IIIa-specific antagonist tirofiban before costimulation with *C. albicans*. Tirofiban is a small-molecule GP IIb/IIIa receptor inhibitor with high affinity and is capable of preventing the binding of fibrinogen and von Willebrand factor to activated GP IIb/IIIa. Tirofiban also reacts with the resting form of GP IIb/IIIa, so it binds to non-stimulated and stimulated platelets (Hagemeyer and Peter, 2010). We found that treatment of quiescent platelets with tirofiban blocked GP IIb/IIIa and reduced the detectable amounts of GP IIb/IIIa on platelets (**Supplemental Figure S4**).We then found that pretreatment of platelets with tirofiban significantly suppressed the expression of P-selectin in a concentration-dependent manner (**Figures 3C–F**). In order to eliminate the interference of fibrinogen in PRP, we confirmed the results by using washed platelets (**Supplemental Figure S5**). We also confirmed the results by pretreatment of washed platelets with blocking antibody for GP IIb/IIIa, which significantly suppressed the expression of P-selectin (**Figures 3G, H**). In addition, pretreatment of platelets with tirofiban almost completely reversed the elevated AKT Ser473 phosphorylation levels caused by *C. albicans* (**Figure 3I**). Taken together, these data suggest that *C. albicans* induces platelet activation in a GP IIb/IIIa-dependent manner.

### Platelets Enhance Human Monocyte Phagocytosis and Expression of Inflammatory Cytokines

**Figure 4:**
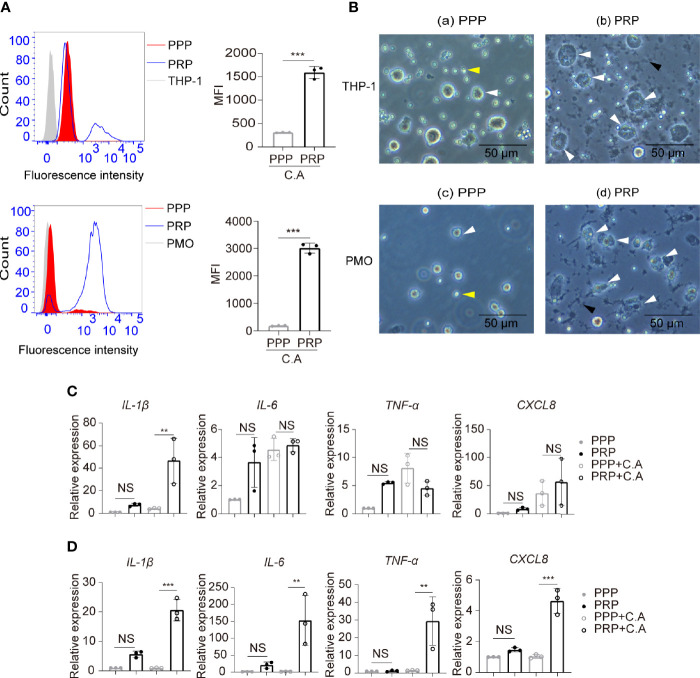
Platelets enhance human monocyte phagocytosis and expression of inflammatory cytokines. THP-1 monocytes or primary human monocytes were incubated with the fluorescent dye FUN-1-labeled *C. albicans* in the presence of PRP or PPP at 37°C for 2 h (3:1, fungi to monocytes; 50:1, platelets to monocytes). **(A)** Phagocytosis of FUN-1-labeled *C. albicans* by THP-1 cells (top column) or primary human monocytes (bottom column) was analyzed by flow cytometry. Untreated THP-1 cells or primary human monocytes were used as a negative control. **(B)** Representative images of THP-1cells **(a, b)** or primary human monocytes **(c, d)** phagocytosing *C. albicans*. White arrows, monocytes or monocytes with intracellular *C. albicans*. Black arrows, platelet aggregation. Yellow arrows, *C. albicans*. **(C, D)** mRNA expression of the inflammatory cytokines *IL-1β*, *IL-6*, *TNF-α* and *CXCL8* by THP-1 cells **(C)** or primary human monocytes **(D)** treated or untreated with *C. albicans* in the presence of PRP or PPP at 37°C for 2h. **P < 0.01, ***P < 0.001 compared with the control by Student’s t test **(A)** or one-way ANOVA followed by Bonferroni’s *post hoc* test **(C, D)**. C.A, *C. albicans*; NS, not significant; PMO, primary human monocytes.

Platelets are capable of promoting phagocytosis and affecting inflammation responses of monocytes-macrophages during bacterial infection (Weyrich et al., 1996; Rodland et al., 2010; Carestia et al., 2019), and the inflammatory cytokines such as IL-1β, IL-6 and TNF-α play a critical role in the outcome of candidiasis by recruiting activated leukocytes (Netea et al., 2015).Therefore, we further explored whether platelets had an effect on monocyte phagocytosis of *C. albicans* and the expression of inflammatory cytokines. We found that compared to PPP, PRP enhanced the capacity of THP-1 monocytes and primary human monocytes to phagocytose FUN-1-labeled *C. albicans* (**Figures 4A, B**). We observed that PRP upregulated the expression of *IL-1β* by THP-1 monocytes (**Figure 4C**) and the expression of *IL-1β*, *IL-6*, *TNF-α* and *CXCL8* by primary human monocytes in the presence of *C. albicans* (**Figure 4D**).

### The Platelet-Regulated Immune Response of Monocytes Is Dependent on GP IIb/IIIa-Mediated Platelet Activation

**Figure 5:**
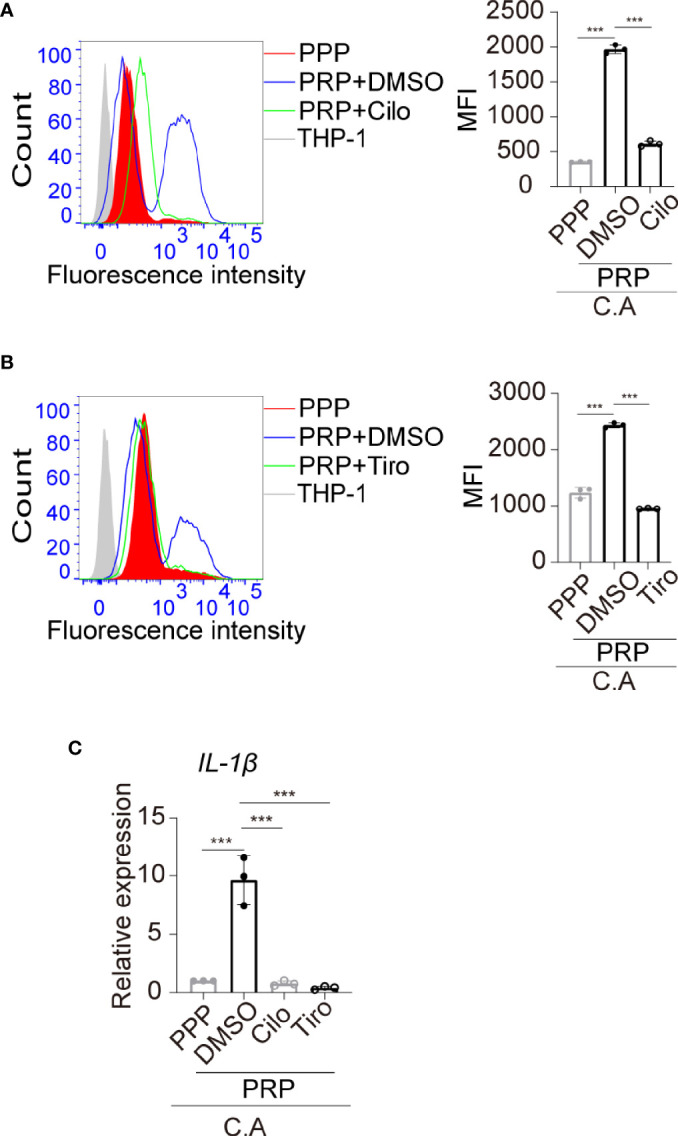
The platelet-regulated immune response of monocytes is dependent on GP IIb/IIIa-mediated platelet activation. **(A)** PRP was pretreated with cilostazol (200 μM) or vehicle control at 37°C for 45 min and then incubated with THP-1 monocytes in the presence of FUN 1-labeled *C. albicans* at 37°C for 2h. The phagocytosis of FUN 1-labeled *C. albicans* by THP-1 monocytes was analyzed by flow cytometry. Untreated THP-1 cells were used as a negative control. **(B)** PRP was pretreated with tirofiban (200 μg/mL) or vehicle control at 37°C for 45 min and then incubated with THP-1 monocytes in the presence of FUN 1-labeled *C. albicans* at 37°C for 2h. The phagocytosis of FUN 1-labeled *C. albicans* by THP-1 monocytes was analyzed by flow cytometry. **(C)** mRNA expression of *IL-1β* was analyzed by qPCR. ***P < 0.001 compared with one-way ANOVA followed by Bonferroni’s *post hoc* test **(A–C)**. C.A, *C. albicans*; Cilo, cilostazol; Tiro, tirofiban.

We found that *C. albicans* induced platelet activation, and platelet aggregations were observed in the monocyte, *C. albicans*, and platelet coculture system (**Figure 4B**, black arrow). Therefore, we further determined whether the elevated phagocytosis activity of monocytes was dependent on platelet activation. Anti-platelet agent cilostazol is a specific and strong inhibitor of PDE3 in platelets, and it diminishes intracellular calcium, causing inhibition of platelet activation and aggregation (Schror, 2002). Cilostazol significantly inhibits platelet activation *in vitro*, and its inhibition effect is much stronger than that of the well known anti-platelet agent aspirin (Ito et al., 2004). We found that treatment of platelets with cilostazol before coculture with THP-1 cells and *C. albicans* removed the elevated phagocytosis effect in PRP-treated THP-1 cells (**Figure 5A**). These data suggest that activated platelets enhance monocyte phagocytosis of *C. albicans*. To elucidate whether GP IIb/IIIa contributes to platelet activation and platelet-mediated phagocytosis of monocytes in this setting, we pretreated platelets with tirofiban before coculture with THP-1 cells and *C. albicans*. We observed that blocking GP IIb/IIIa receptors on platelets inhibited platelet aggregation (**Supplemental Figure S6**) and removed the elevated phagocytosis effect in PRP-treated THP-1 cells (**Figure 5B**). Additionally, pretreatment of platelets with cilostazol or tirofiban removed the elevated expression of *IL-1β* induced in THP-1 cells treated with *C. albicans* (**Figure 5C**).

## Discussion

The data described in this study indicate that (*i*) *C. albicans* induces platelet activation through the PI3K-AKT signaling pathway; (*ii*) *C. albicans* induces platelet activation in a GP IIb/IIIa-dependent manner, independent of TLR2 and TLR4; and (*iii*) platelets enhance human monocyte phagocytosis and upregulate the expression of inflammatory cytokines, which are dependent on platelet activation mediated by GP IIb/IIIa.

Our experiments showed that live *C. albicans* induced platelet activation *in vitro*. We also confirmed the effect using HK *C. albicans*, which did not proliferate and allowed us to avoid changes in the ratio between yeasts and platelets. Previous studies on whether *C. albicans* induces platelet activation are limited and controversial. Similar to our own findings, Robert et al. and Eberl et al. described that attachment of platelets to *C. albicans in vivo* and *in vitro*, and these interactions resulted in activation-dependent morphological changes (Robert et al., 1996; Robert et al., 2000; Eberl et al., 2019). However, Eberl et al. also showed that the complete platelet population (most were nonadhered platelets) was not activated *in vitro* (Eberl et al., 2019). We noticed that their experimental conditions were similar to ours since we both used the *C. albicans* SC5314 strain to stimulate PRP. Notably, they used higher MOI but shorter incubation times. Unlike the platelet agonist thrombin, which is often used as a positive control, *C. albicans*-induced platelet activation was milder. Therefore, we observed the effects for a longer time. Other studies have reported the inability of *C. albicans* to activate platelets or induce their aggregation (Willcox et al., 1998; Schultz et al., 2020). We analyzed their experimental settings and found that compared to our experimental conditions, they had used either shorter incubation times or the same incubation time but lower MOI. Overall, we conclude that incubation time and MOI may be the main causes of diverse results *in vitro*. Indeed, when the body is infected with *C. albicans*, the MOI is variable, depending on the body’s resistance, duration of infection, etc. Combined with our results and those of others, we speculate *C. albicans* causes platelet activation in systemic infection when MOI and duration of infection reach a certain threshold.

Our results showed that *C. albicans* markedly elevated platelet secretion of PF4 and CCL5, which exerted direct activities against a variety of pathogens, including *C. albicans* (Tang et al., 2002; Yeaman, 2014). We found that the expression of β-defensin-2, another antimicrobial peptide, was slightly elevated in *C. albicans*-treated platelets (but not significantly elevated in those treated with live *C. albicans*). We also found that *C. albicans* induced platelets to release low concentrations of TNF-α and CXCL8. Given the large number of circulating platelets, elevated TNF-α and CXCL8 levels may crucially affect the outcome of candidemia. Ali et al. reported that IL-1β was released by thrombin-activated platelets and mediated host defense against *Staphylococcus aureus* (Ali et al., 2017). Lindemann et al. showed that activated platelets synthesized IL-1β by translating IL-1β mRNA into protein during fibrin clot formation (Lindemann et al., 2001). However, we found that *C. albicans*-activated platelets did not exhibit increased release of IL-1β or expression of IL-1β mRNA (data not shown).

Previous literature has indicated that GP IIb/IIIa recognized the fungus *Mucor circinelloides* and a variety of viruses, such as dengue virus and hantavirus, leading to platelet activation and aggregation (Ghuman et al., 2019; Gautam et al., 2020). However, studies on GP IIb/IIIa-mediated interactions between *C. albicans* and platelets are limited. Our data showed that GP IIb/IIIa but not TLR2 or TLR4 was receptor of *C. albicans* that mediated platelet activation. Pretreatment of platelets with tirofiban at a high concentration (200 μg/mL) almost reversed *C. albicans*-induced P-selectin expression and AKT Ser473 phosphorylation in platelets. Pretreatment of platelets with anti-GP IIb/IIIa blocking antibody also reversed *C. albicans*-induced P-selectin expression. Thus, GP IIb/IIIa may be the specific receptor that mediates the interactions between *C. albicans* and platelets. This finding was consistent with our observations that different concentrations of TLR2- and TLR4-neutralizing antibodies did not block *C. albicans*-induced platelet activation and that TLR2 and TLR4 agonists did not induce platelet activation as *C. albicans* did. In agreement, previous literature has indicated that *C. albicans* inhibited the binding of fibrinogen and PAC-1, two ligands of GP IIb/IIIa, to thrombin-stimulated platelets (Bertling et al., 2010). The current study provides a theoretical explanation for this finding, which may result from the occupation of GP IIb/IIIa by *C. albicans*. Our data also showed that the binding of FITC-PAC-1 did not vary on HK *C. albicans*-treated and control platelets. Though *C. albicans* could induce platelet activation and therefore might increase the PAC-1binding to activated GP IIb/IIIa, *C. albicans* binding to GP IIb/IIIa might inhibit the PAC-1binding, leading to an undifferentiated result.

Previous literature has indicated elevated Stat3 phosphorylation on Tyr705 in collagen-activated platelets (Zhou et al., 2013). Although Stat3 Tyr705 phosphorylation was elevated in HK *C. albicans*-treated platelets (but not significantly in live *C. albicans*-treated platelets), the phosphorylation level was not remarkable. PI3K/Akt is the most widely investigated signaling pathway in platelet activation and mediates platelet apoptosis (Guidetti et al., 2015; Chen et al., 2018). However, there are few data available on the role of the PI3K/AKT pathway in *C. albicans*-mediated platelet activation. Our data showed that AKT Ser473 phosphorylation levels were significantly elevated in *C. albicans*-treated platelets. Moreover, the PI3K inhibitor LY294002 inhibited AKT Ser473 phosphorylation, indicating that PI3K was upstream of AKT in this system. LY294002 and MK2206 markedly reduced P-selectin expression on platelets, confirming that the PI3K/Akt signaling pathway mediated *C. albicans*-induced platelet activation.

Monocytes play an important role in the innate immune response to defend the host against *C. albicans*. On the one hand, monocytes are recruited to *C. albicans*-infected tissue, where they differentiate into inflammatory macrophages (Netea et al., 2015); on the other hand, monocytes are capable of inhibiting *C. albicans* germination and phagocytosing *C. albicans* (Smeekens et al., 2011). However, scarce data have been reported regarding platelet-monocyte interactions after *C. albicans* stimulation. Our data showed that platelets enhanced THP-1 monocyte and primary human monocyte phagocytosis of *C. albicans* and expression of inflammatory cytokines, which indicated their indirect role in the efficient clearance of *C. albicans*. Cilostazol is a specific inhibitor of PDE3 in platelets, and potently inhibit platelet activation and aggregation *in vitro* (Ito et al., 2004). Our data showed that cilostazol removed the elevated phagocytosis effect and expression of inflammatory cytokine in PRP-treated THP-1 cells. These data suggest that platelets under activated condition regulate immune response of monocytes rather than platelets under steady-state condition. Tirofiban is a GP IIb/IIIa-specific antagonist and is capable of blocking GP IIb/IIIa-mediated platelet activation and aggregation (Fisher et al., 1999; Tummala and Rai, 2021). Our data showed that tirofiban inhibited platelet aggregation and removed the elevated phagocytosis effect and expression of inflammatory cytokine in PRP-treated THP-1 cells. These data suggest that GP IIb/IIIa mediates platelet activation in the platelet, *C. albicans*, and THP-1 cell coculture system, and therefore contributes to platelet-regulated immune response of monocytes. These findings were consistent with previous studies, showing that activated or aggregated platelets facilitated the capture and phagocytosis of bacteria by macrophages, as well as signaled chemokine synthesis by monocytes (Weyrich et al., 1996; Ali et al., 2017; Carestia et al., 2019). Studies have described the crosstalk between platelets and monocytes, depending on the cellular contact between platelet GPIb and monocyte CD11b or platelet P-selectin and monocyte P-selectin glycoprotein-1 (Weyrich et al., 1996; Carestia et al., 2019). Further studies are required to elucidate the crosstalk of platelets and monocytes under the settings of *C. albicans* infection.

Taken together, our findings suggest that activated platelets play a role in innate immune responses against *C. albicans* infection. Platelet-targeted therapy might be a potential option for *C. albicans* infection.

## Data Availability Statement

The raw data supporting the conclusions of this article will be made available by the authors, without undue reservation.

## Ethics Statement

The studies involving human participants were reviewed and approved by Institute of Dermatology, Chinese Academy of Medical Science (CAMS) & Peking Union Medical College and Jiangsu Province Blood Center (NO.2017-KY-022). The patients/participants provided their written informed consent to participate in this study.

## Author Contributions

LZ performed and analyzed the experiments and drafted the manuscript. XC, QC, and ML designed the experiments and reviewed the manuscript. ZD assisted in analyzing the experiments. YH assisted in the *C. albicans* staining. DT gathered and processed human platelets from volunteers. All authors contributed to the article and approved the submitted version.

## Funding

This work was supported by the CAMS Innovation Fund for Medical Science (2017-I2M-1-017), the National Natural Science Foundation of China (81773338), and the Nanjing Incubation Program for the National Clinical Research Center [2019060001].

## Conflict of Interest

The authors declare that the research was conducted in the absence of any commercial or financial relationships that could be construed as a potential conflict of interest.

## Publisher’s Note

All claims expressed in this article are solely those of the authors and do not necessarily represent those of their affiliated organizations, or those of the publisher, the editors and the reviewers. Any product that may be evaluated in this article, or claim that may be made by its manufacturer, is not guaranteed or endorsed by the publisher.

## Abbreviations

*A. fumigates*, *Aspergillus fumigates*; ANOVA, analysis of variance; *C. albicans*, *Candida albicans*; ELISA, enzyme-linked immunosorbent assay; GP IIb/IIIa, Glycoprotein IIb/IIIa; HK *C. albicans*, heat-killed *Candida albicans*; MFI, mean fluorescence intensity; MOI, multiplicity of infection; PBS, phosphate-buffered saline; PF4, platelet factor 4; PMPs, platelet microbicidal proteins; PPP, platelet-poor plasma; PRP, platelet-rich plasma; TLR, toll-like receptor.
