# Supplementary material for: GP IIb/IIIa-Mediated Platelet Activation and Its Modulation of the Immune Response of Monocytes Against Candida albicans
Source: Front Cell Infect Microbiol. 2021 Dec 6;11:783085. doi: 10.3389/fcimb.2021.783085 (PMC8685400; doi:10.3389/fcimb.2021.783085)
Supplement: Supplementary file 1 [file DataSheet_1.docx]

**Supplementary figures**

**
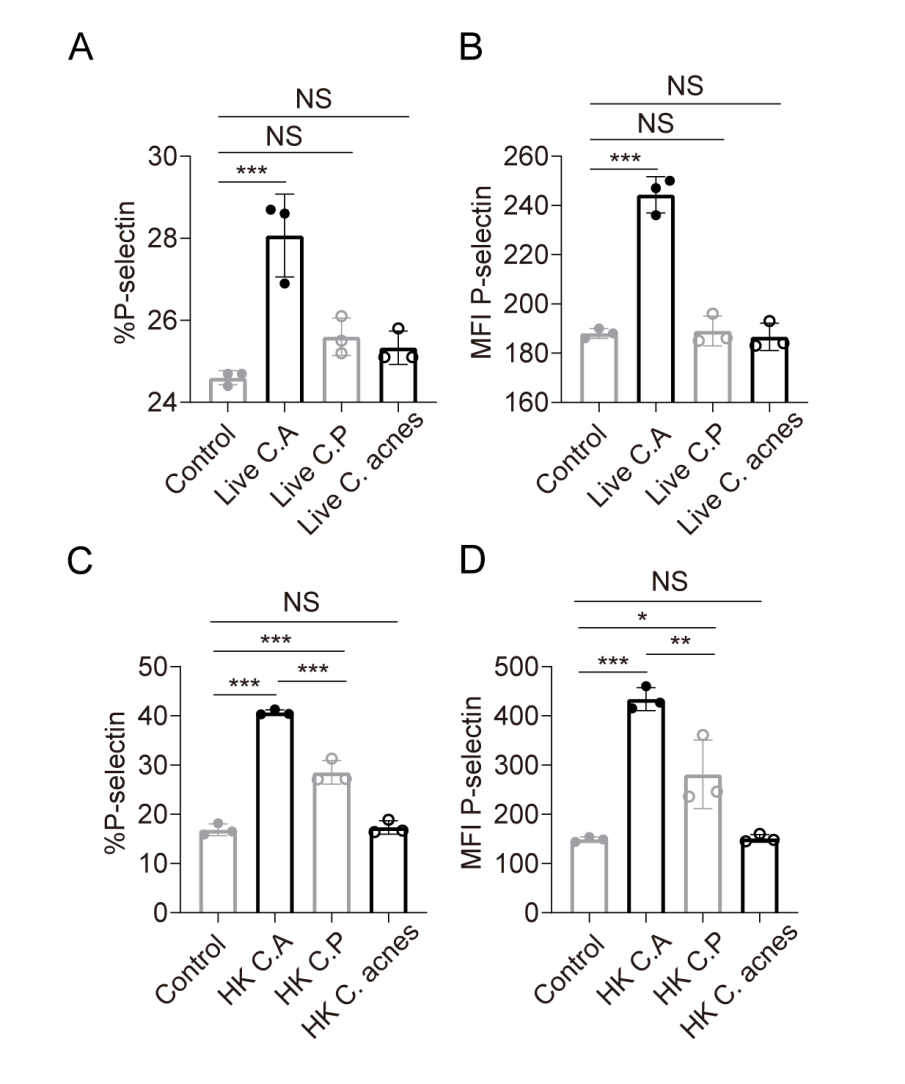
**

**Supplemental figure 1. *C. albicans-*treated platelets express more P-selectin than *C. parapsilosis-* or *C. acnes-*treated platelets.**

(A-D) Platelet-rich plasma (PRP) was incubated with live *C. albicans*, *C. parapsilosis* and *C. acnes* for 2 h (A-B) or heat-killed (HK) *C. albicans*, *C. parapsilosis* and *C. acnes* for 6 h (C-D) at a multiplicity of infection (MOI) of 0.01. PRP incubated with equal volume of PBS was used as control. Cells were fixed, stained for the general platelet marker CD41 and the activation marker P-selectin and analyzed using flow cytometry. The percentages (A and C) and mean fluorescence intensity (MFI) (B and D) of P-selectin-positive cells were calculated. Data are expressed as the mean ± SD determined by one-way ANOVA followed by Bonferroni’s post hoc test; **P* < 0.05, ***P* < 0.01, ****P* < 0.001. C.A, *C. albicans*. C.P, *C. parapsilosis.* HK, heat-killed. NS, not significant.


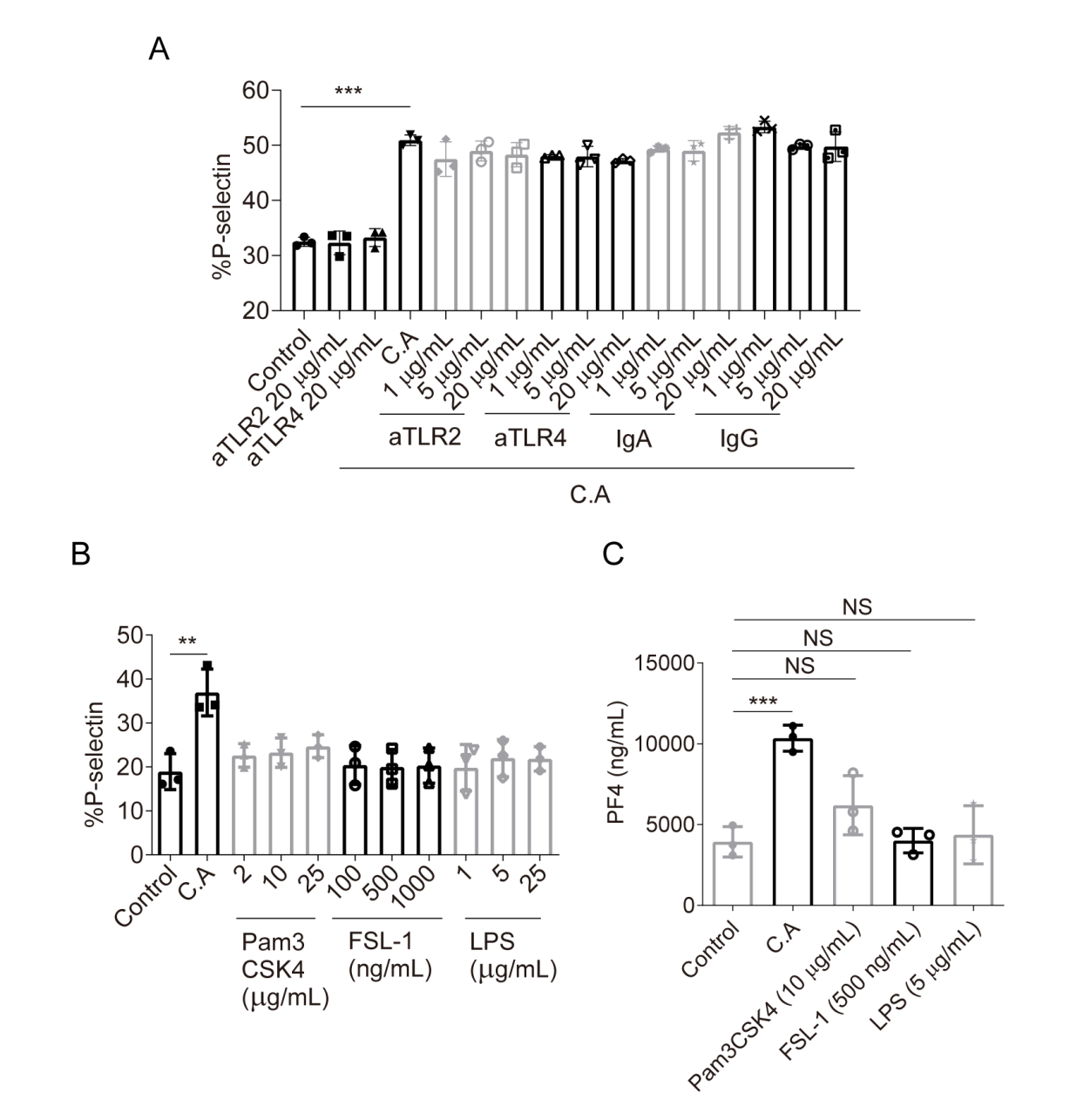


**Supplemental figure 2. *C. albicans* induces platelet activation independent of TLR2 and TLR4.**

(A) Human platelets were pretreated with anti-TLR2 and anti-TLR4 neutralizing (1, 5, or 20 μg/mL) or vehicle control at 37 °C for 45 min and then incubated with *C. albicans* at 37 °C for 2 h. The percentages of P-selectin-positive cells were calculated. (B-C) Platelets were stimulated with the TLR2 agonists Pam3CSK4 (2, 10, or 25 μg/mL) and FSL-1 (0.1, 0.5, or 1 μg/mL) and the TLR4 agonist LPS (1, 5, or 25 μg/mL) at 37 °C for 6 h. HK *C. albicans* was used as a positive control. The percentages of P-selectin-positive cells were calculated (B), and the levels of PF4 in the coculture suspension were determined by ELISA. Data are expressed as the mean ± SD determined by one-way ANOVA followed by Bonferroni’s post hoc test; ***P* < 0.01, ****P* < 0.001. C.A, *C. albicans*. NS, not significant.


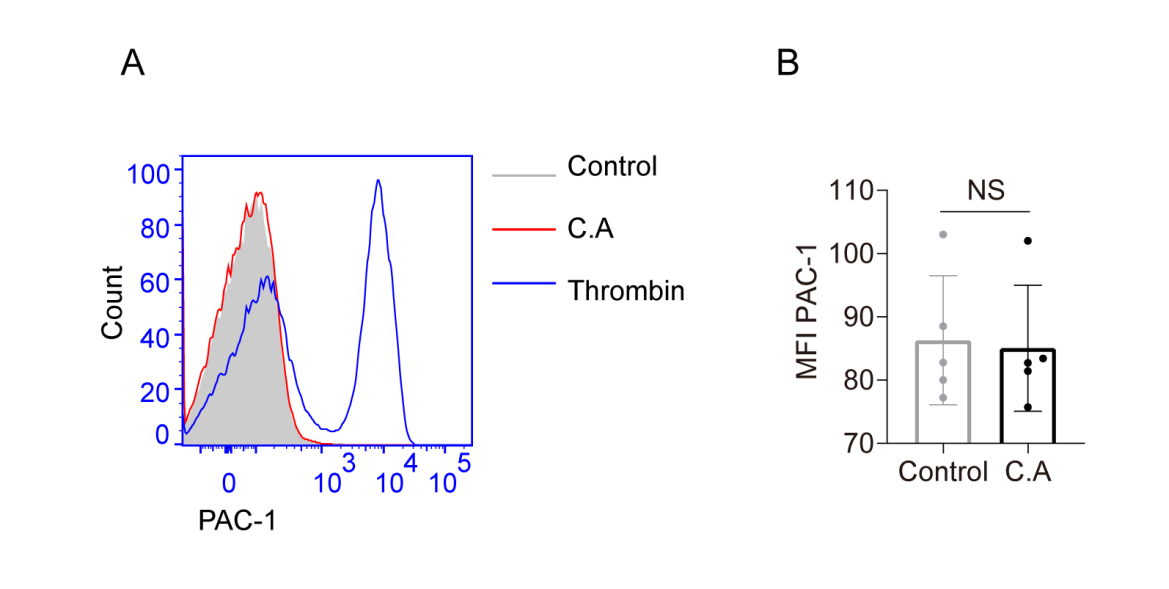


**Supplemental figure 3. The binding of PAC-1 in *C. albicans-*treated and control platelets.**

(A-B) Washed platelets were incubated with live *C. albicans* or equal volume of PBS for 2 h at an MOI of 0.01. Washed platelets treated with thrombin (0.5 U/mL) for 5 min were used as a positive control. Cells were stained for the general platelet marker CD41 and FITC-coupled antibody PAC-1 and analyzed using flow cytometry. Flow cytometry histograms (A) are shown. The MFI (B) of PAC-1-positive cells was calculated. Data are expressed as the mean ± SD determined by Student’s t test. C.A, *C. albicans*. NS, not significant.

**
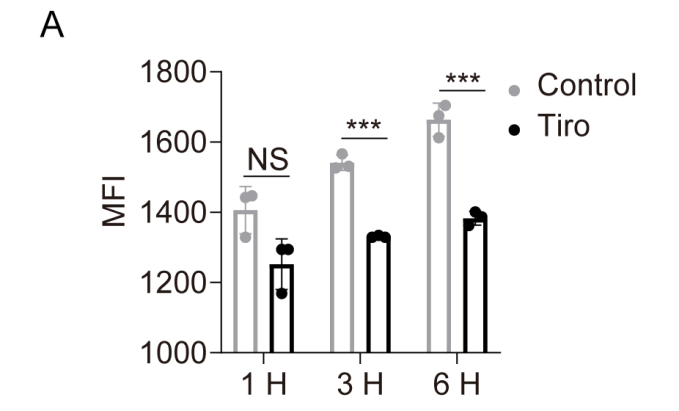
**

**Supplemental figure 4. Tirofiban reduces the detectable amounts of GP IIb/IIIa on quiescent platelets.**

(A) PRP was treated with the GP IIb/IIIa-specific antagonist tirofiban at 200 μg/mL or vehicle control DMSO at 37 °C for 1 h, 3 h, and 6 h. The expression of GP IIb/IIIa (CD41/CD61) was detected by flow cytometry using a PE-Cy7-conjugated anti-CD41a antibody. The MFI of GP IIb/IIIa-positive cells was calculated. Data are expressed as the mean ± SD; ****P* < 0.001, compared with the vehicle control via Student’s *t* test. NS, not significant. Tiro, tirofiban.


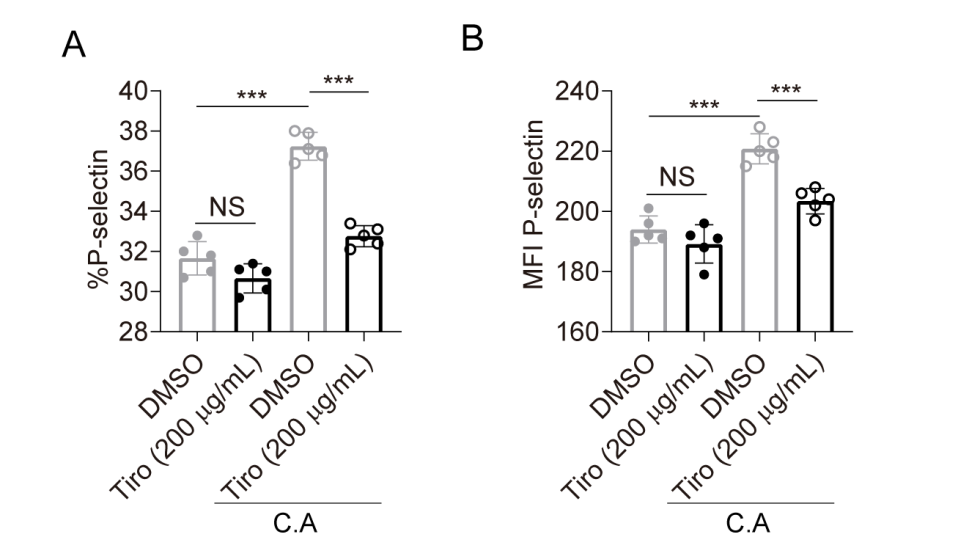


**Supplemental figure 5. Tirofiban suppresses P-selectin expression on** ***C. albicans-*treated washed platelets.**

(A-B) Washed platelets were pretreated with tirofiban at 200 μg/mL or vehicle control DMSO at 37 °C for 45 min and then incubated with live *C. albicans* at 37 °C for 2 h. The percentages (A) and MFI (B) of P-selectin-positive cells were calculated. Data are expressed as the mean ± SD determined by one-way ANOVA followed by Bonferroni’s post hoc test; ****P* < 0.001. C.A, *C. albicans*. NS, not significant. Tiro, tirofiban.

**PRP+Tiro**

**PRP+DMSO**


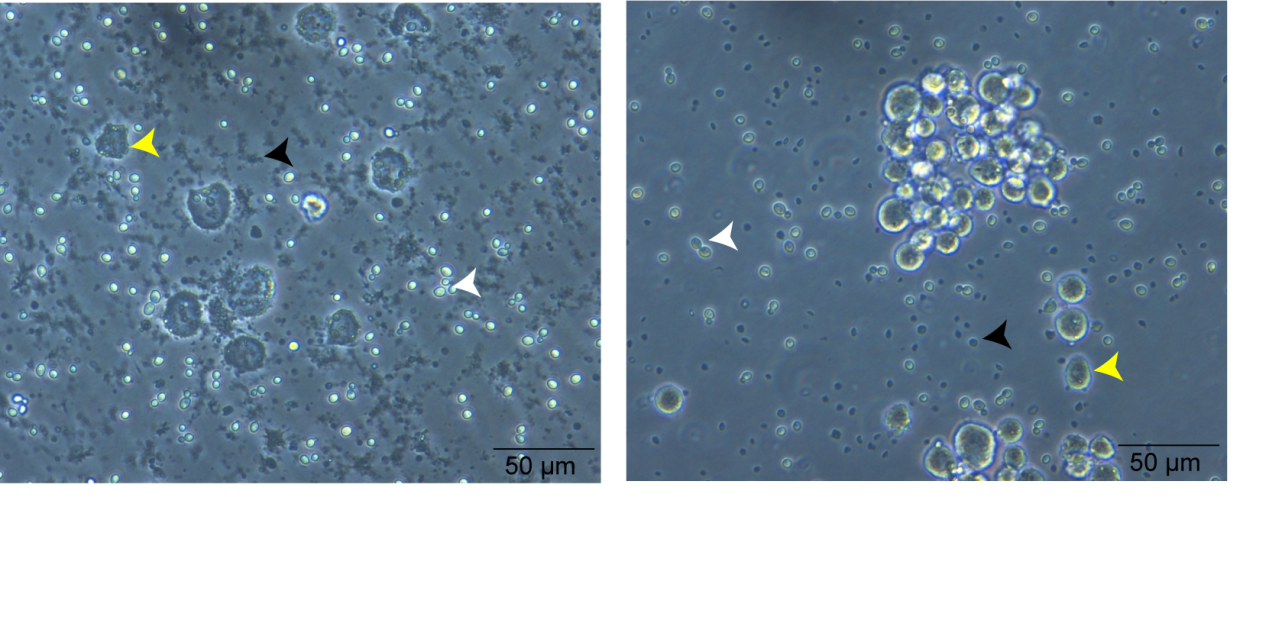
**Supplemental figure 6.** **The** **GP IIb/IIIa-specific antagonist tirofiban inhibits platelet aggregation.**

**B**

**A**

(A-B) PRP was pretreated with tirofiban (200 μg/mL) or vehicle control at 37 °C for 45 min and then incubated with THP-1 monocytes in the presence of FUN 1-labeled *C. albicans* at 37 °C for 2 h. Pretreatment of platelets with tirofiban (200 μg/mL) inhibited platelet aggregation (B). White arrow, *C. albicans*. Black arrow, platelet aggregation (A) or platelet (B). Yellow arrow, THP-1 monocyte. Tiro, tirofiban.

**Supplementary methods**

***Candida parapsilosis* (*C. parapsilosis*) and *Cutibacterium acnes* (*C. acnes*) Cultivation**

*C. parapsilosis* C4F (from the China Medical Fungus Culture Collection Center) was grown for 12 h at 30 °C in yeast extract-peptone-dextrose (YPD) broth. *C. parapsilosis* was then collected by centrifugation at 2000×*g* for 5 min. Following centrifugation, *C. parapsilosis* was resuspended in 30 mL of phosphate-buffered saline (PBS) and washed twice. Yeast cells were enumerated using a hemocytometer and then diluted with PBS to the desired working concentrations. Then the yeast cells were used in platelet treatment. In some experiments, after being washed and diluted with PBS, *C. parapsilosis* was heat killed immediately in an 85 °C water bath for 10 min. *C. acnes* was purchased from the Guangdong Microbial Culture Collection Center (GDMCC, Guangzhou, China, GDMCC 1.162). The isolates were retrieved from its frozen glycerol stocks and colonies were achieved following incubation for 72 h to 96 h at 37 °C in brain heart infusion (BHI) broth under anaerobic conditions. Bacteria were washed and resuspended in sterile PBS. Then bacteria were diluted with PBS to the desired working concentrations. In some experiments, *C. acnes* was heat killed in a 100 °C water bath for 20 min. The conditions of platelet stimulation with *C. parapsilosis* and *C. acnes* were the same with that with *C. albicans.*

**Binding of PAC-1**

Washed platelets were incubated with live *C. albicans* or equal volume of PBS for 2 h at a multiplicity of infection (MOI) of 0.01. Washed platelets treated with thrombin (Sigma-Aldrich, T6884, 0.5 U/mL) for 5 min were used as a positive control. Platelets were then diluted with modified Tyrode's solution (Beijing Solarbio Science and Technology Co., Ltd., Beijing, China) to 10^6^/100 μL. Platelets were stained with 1:200-diluted CD41a-PE-Cy7 (Thermo Fisher, 25-0419-42, clone HIP8) and 1:40-diluted FITC-coupled antibody PAC-1 (Thermo Fisher, MA5-28564, clone PAC-1). The samples were incubated in a dark environment for 30 min at room temperature. After incubation, 200 μL of PBS was added to each tube and the samples were analysed immediately on a flow cytometer (BD FACSVers).

**Quiescent Platelets Treatment with Tirofiban**

PRP was treated with the GP IIb/IIIa-specific antagonist tirofiban at 200 μg/mL or vehicle control DMSO at 37 °C for 1 h, 3 h, and 6 h. Platelets were then diluted with PBS to 10^6^/100 μL. Platelets were stained with 1:200-diluted CD41a-PE-Cy7 in a dark environment for 30 min at room temperature (avoiding violent vibration). After incubation, platelets were washed with 2 mL ice-cold PBS and the samples were analysed immediately on a flow cytometer (BD FACSVers).

**Washed Platelet Treatment with Tirofiban and *C. albicans***

After washed platelet preparation, platelets were pretreated with tirofiban (200 μg/mL) or vehicle control DMSO at 37 °C for 45 min and then costimulated with live *C. albicans* at 37 °C for 2 h. Cells were fixed, stained for the general platelet marker CD41 and the activation marker P-selectin and analyzed using flow cytometry.
